# Employment-related difficulties and distressed living condition in patients with hepatitis B virus: A qualitative and quantitative study

**DOI:** 10.1186/s12889-017-4416-3

**Published:** 2017-06-12

**Authors:** Taeko Oka, Hiroaki Enoki, Yukari Tokimoto, Teruaki Kawanishi, Meguru Minami, Takahiro Okuizumi, Kiyohiko Katahira

**Affiliations:** 1 Human Environment University, 3-6, Hanazonomachi, Matsuyama, Ehime Japan; 2 Research Institute of Clinical and Social Pharmacy, 4-510-1 Takano, Misato, Saitama, Japan; 3 Osaka University of Human Sciences, 1-4-1 Shojaku, Settsu, Osaka, Japan; 4 Sapporo Ryokuai Hospital, Hokkaido Health Coop, 1-6-30 Kitano 1-Jo, Kiyota-ku, Sapporo, Japan; 5 Koendori Law Office, Hokuseiodori Bldg., 2F., Odori West 13-4, Chuo-ku, Sapporo, Hokkaido Japan

**Keywords:** Group vaccination, HBV, Employment difficulties and poverty, Distressed living conditions, KJ method

## Abstract

**Background:**

In Japan, an estimated 400,000 people have the hepatitis B virus (HBV), many of whom were infected as a result of group vaccinations. People with HBV face many challenges, including disease progression, employment-related difficulties, and increased medical expenses. The relationship between HBV victims’ daily life suffering and poverty associated with HBV-related employment changes has not been examined. We aimed to clarify the employment-related hardships experienced by Japanese HBV victims, and the relationships between these hardships and daily life suffering, including poverty, through qualitative and quantitative analyses.

**Methods:**

The study population comprised 11,046 people infected with HBV via group vaccination who filed lawsuits in Japan’s District Courts by 2014. First, we conducted a qualitative study (2013) using the KJ method, with 107 participants (68 men, mean age 58.9 years; 39 women, mean age 55.3 years). Semi-structured interviews were conducted covering participants’ current condition, treatment, medical expenses, and life difficulties (employment- and family-related problems). In 2014, we conducted a quantitative study. We mailed questionnaires to the entire study population, investigating the topics covered in the interviews (response rate 60.1%). Daily life suffering was determined by responses to the question “What do you think about your everyday life situation?” We performed binomial logistic regression analyses to verify the relationships between daily life suffering and disease, employment, and income status.

**Results:**

Interview data were integrated into seven islands: intention to work, lack of understanding of HBV in the workplace, inability to buy life insurance, burden due to medical expenses, life failure, dissatisfaction with the system, and wishing for life balance. The quantitative analyses showed significant positive correlations between daily life suffering and liver cancer (odds ratio [OR] 1.47, 95% confidence interval [CI]: 1.00–2.17, *p* < 0.05), being a part-time/casual employee (OR 1.46, 95% CI: 1.11–1.92 *p* < 0.01), and an income below the national average (*p* < 0.01).

**Conclusion:**

We qualitatively and quantitatively demonstrated that employment-related hardships and daily life suffering are prevalent in people with HBV. Their likelihood of experiencing distress in daily life increases with increasing medical expenses, insecure employment status (e.g., job loss) attributable to HBV, and the resulting poverty.

## Background

In Japan, an estimated 400,000 people are infected with the hepatitis B virus (HBV), a viral hepatitis that is one of the most widespread infections in the country, mainly as the result of group vaccinations. The Japanese Ministry of Health, Labour and Welfare (2015) had to provide relief for these patients and implement permanent measures against HBV [1]. Research challenges that are particularly urgent include developing understanding of the difficulties faced by HBV victims in their daily lives and their support needs, and proposing appropriate welfare policies.

The lawsuit over drug-induced hepatitis B started in 1989, when five patients filed a complaint with the Sapporo District Court about the causal relationship between the group vaccination they received in infancy and HBV infection damage [2, 3]. Seventeen years later, the plaintiffs won the lawsuit in the Supreme Court [4, 5]. After the preparation of a “Basic Agreement” on the lawsuit over drug-induced HBV in 2011 and apologies from the Japanese government, the Ministry of Health, Labour and Welfare established a “Committee on the Verification and the Recurrence Prevention of the infection spread of hepatitis B caused by the group vaccination” (hereafter the Verification Committee) in 2012. The Verification Committee prepared a report stating that “the challenges in the national system, the system framework, and the specific operation caused the infection spread of hepatitis B in the lawsuit over drug-induced hepatitis B” [6].

That report noted that in contrast to European countries and the US which have thorough vaccination safety controls, group vaccination was performed in Japan. In addition, it was found that a combination of factors, such as group vaccination performed on the same date in the same site, obligatory vaccination (with fines) for the people, continuous use of injection devices for 40 years (from 1948 to 1988), and non-compliance with international standards set by the World Health Organization (WHO) expanded the damage.

Vaccination is performed to secure active immunity of children in infancy whose immunity functions are immature and who are in the phase of receiving infection prevention activities. Children usually receive the vaccination individually in medical institutions. However, in Japan, many people were gathered in one place (such as a community hall or school) and group vaccination was performed as an efficient measure against infection. After the Second World War, more than 700,000 patients had variola, diphtheria, typhoid, paratyphoid, epidemic typhus, tuberculosis, and polio, and immediate prevention of the spread of epidemics across Japan was necessary by decreasing the number of epidemic-related deaths, protecting human lives, and preventing economic loss. Vaccination efforts were directed by the general headquarters, and under the 1948 Preventive Vaccination Act, vaccination was compulsory and obligatory.

Vaccination directives were that the injection needle used for vaccination needed to be replaced with a needle sterilized by dry or moist heating for each person who received an injection, an injection barrel smaller than 5cm^3^ had to be used, and a newly sterilized injection needle used each time the barrel was filled with vaccine [7]. However, at that time, it was thought that if the injection needle was replaced it was not necessary to replace the injection barrel to prevent infections. Therefore, although the needle should have been sterilized and replaced for each person, needles were actually continuously used. For the tuberculin reaction test, in addition to provisions for the sterilization of injection devices and injection needles, repetition of the operation and continued use of needles until the tuberculin in an injection device ran out without replacing the needle itself was allowed. Under these conditions, 60–150 million vaccinations were performed annually until the 1950s.

For group vaccinations, one doctor vaccinated 80 people per hour for variola, 100 people for pertussis, 120 people for the tuberculin reaction test, and 150 people for diphtheria, typhoid, paratyphoid, epidemic typhus, and so forth. The large number of people that each doctor had to vaccinate made replacing the injection barrel for each person difficult. Therefore, a small amount of body fluids flowed back into continuously used injection needles or barrels. There is a risk that 0.0004 ml of contaminated blood used for people with the virus might also cause infection, meaning HBV infection spread due to group vaccination [8].

Since the 1940s, it has been recognized that people may become infected with hepatitis by blood transfusion or plasma injection, and that viruses may be transmitted by insufficient sterilization of injection needles or barrels. The WHO proposed “replacement of injection needles and barrels in vaccination” in 1953 [9], but Japan did not follow this recommendation. Finally, in the 1987 WHO proposal for developing countries [10], it became a written rule that injection needles and barrels be replaced for each person who received vaccination. Therefore, people started using clean injection needles and barrels. However, before this 1987 WHO directive, many local communities had already started using disposable injection devices and fewer people were continuously using needles and barrels.

Few studies on HBV infection damage due to group vaccination are available, and limited qualitative studies [11, 12] have reported on the results of the damage survey conducted by the Verification Committee. One study revealed serious problems such as inadequate medical institution reactions when the HBV infection was found; social exclusion in medical institutions, public organizations, or offices; and an increasing number of patients who died of progression or exacerbation of hepatitis. This is of particular concern as patients with serious hepatic cirrhosis or liver cancer are required to visit or stay in a hospital for 20 ~ 30 days each year.

In addition, some reports have highlighted the difficulties in daily life faced by people infected with viral infections. For example, people infected with HIV cannot avoid delay in notification of the infection by the doctor [13], health damage such as opportunistic infection and liver diseases [14], and life-activity self-limitation due to discrimination anxiety [15]. People infected with the hepatitis C virus (HCV) also suffer physical and mental pain, fear of prejudice and discrimination [16], and difficulties and economic burden related to continued long-term care. The difficulties faced by people who are infected with hepatitis are similar to those faced by people with leprosy, including misunderstanding about infectivity (leprosy is less infectious), extreme social exclusion due to the disease becoming serious [17, 18], and self-stigma [19]. However, at the same time, cases have been reported in which those concerned and their supporters expended effort for the relief and prevention of recurrence of health damage, including the formation of self-help groups for people infected with HIV [20], an epoch-making care promotion by the HIV-positive people campaign [21], and “beyond silence,” the lawsuit over drug-induced HBV [22].

The Verification Committee report includes the results of a survey of victims infected with HBV (1311 respondents) and bereaved families (103 respondents) who came to a settlement. This states that the victims had no choice but to change jobs (retirement, reallocation, and job transfer, 24.1%), take pay cuts (70%), or were refused entry to private insurance (27.3%) [1] during their struggle with the disease.

Despite the acknowledgement of these struggles, the relationship between the suffering experienced by HBV victims in their everyday life and the poverty associated with resulting employment changes has not been analyzed. Therefore, through qualitative and quantitative analyses, we aimed to clarify the employment-related hardship experienced by Japanese HBV victims, and determine the relationship between their everyday suffering, including the resulting poverty.

## Methods

### Participants

In cooperation with the nationwide HBV plaintiff group and its lawyers, the present study targeted 11,046 people who were infected with HBV via group vaccination and had filed a lawsuit in District Courts across Japan by 2014. A qualitative study using the KJ method extracted common characteristics of these people (e.g., cause of infection: vaccination or mother-to-child transmission, pathology, age, sex, region, experience of discrimination, difficulty working, and family problems caused by infection), and selected 107 people who cooperated with requests for investigation. A postal quantitative questionnaire study targeting members of the nationwide HBV plaintiff group (including the qualitative study participants) received responses from 6525 people (response rate: 60.1%). Participants’ characteristics are shown in Table 1.

**Table 1:** The attributes and answers to questionnaire of HBV Victims

	Mean/Number	SD/%
	Mean	SD
Age		
	56.8	10.4
	Number	%
Sex		
Male	4077	62.0
Female	2504	38.0
Everyday Life Situations		
Suffering	3073	47.1
Not suffering	3452	52.9
Settlement acceptance		
Accepted	3791	59.1
Not accepted	2624	40.9
Work-related problems		
Yes	2337	47.1
No	2621	52.9
Debt-related problems		
Yes	1911	38.5
No	3047	61.5
Does the present condition of your disease fall into the following items?		
	Number	%
Asymptomatic carrier		
Yes	1803	28.3
No	4573	71.7
Chronic hepatitis		
Yes	3313	52
No	3063	48
Cirrhosis		
Yes	819	12.8
No	5557	87.2
Liver cancer		
Yes	692	10.9
No	5684	89.1
Does the present employment forms of yours fall into the following items?		
	Number	%
Regular employee (full-time etc.)		
Yes	1874	7.3
No	4777	92.7
Non-regular employee (dispatch, contract, temporary worker, etc.)		
Yes	484	7.3
No	6167	92.7
Part-time/casual employee		
Yes	740	11.1
No	5911	88.9
Unemployed		
Yes	1476	22.2
No	5175	77.8
Does the latest yearly income of yours fall into the following items?		
	Number	%
0-1 million yen		
Yes	424	7.7
No	4574	92.3
1-2 million yen		
Yes	928	16.9
No	4574	83.1
2-3 million yen		
Yes	1280	23.3
No	4222	76.7
3-4 million yen		
Yes	1114	20.2
No	4388	79.8
4-5 million yen		
Yes	703	12.8
No	4799	87.2
5-6 million yen		
Yes	510	9.3
No	4992	90.7

### Data collection

We conducted semi-structured interviews for our qualitative study using the KJ method. Of the interview participants, 68 were male and 39 were female (average age: 58.9 and 55.3 years, respectively). Each interview was conducted by 1–4 researchers with a single interviewee, and lasted 90–120 min. An interview guide was used, with questions designed according to the study’s goals and reference materials such as reports of Verification Committee meetings and surveys of patients with HCV [23]. The questions covered the patient’s situation immediately after infection and their current condition; medical institutions, treatment, and treatment expenses paid by the patient; life difficulties such as work- and family-related problems; and requests for government/social aid. The interviews were conducted once and there was no follow-up.

The quantitative questionnaire was administered by mail. When designing the questions, in addition to integrating insights gained through the KJ method, we used measurement scales as used in the reports of Verification Committee meetings, the Comprehensive Survey of Living Conditions, and a quality of life index (i.e., the SF-36). The questions investigated the same topics covered in the interviews.

### Analyses

We attempted to clarify the employment-related hardships experienced by patients with HBV from both subjective and objective perspectives (i.e., individual-level causation and statistical relationships). Subjective and objective factors were analyzed using qualitative and quantitative methods, respectively.

Qualitative analyses were performed with interview data using the KJ method [24]. Based on the purpose of the research, relevant descriptions were extracted from the word-for-word record list created by consensus between investigators, and transferred to 925 KJ labels. Then, a narrow-sense KJ method was conducted via group work with the 20 selected original labels by multistage pickup. To ensure the credibility and validity of the qualitative research, the analyses were supervised by Ms. Akiko Kawakita, who hosts the Mushin-kan—KJ method education/training course, which teaches an interview method to obtain good-quality data without asking leading questions. In addition, as a supervisory function of our research process, we repeatedly confirmed with researchers, doctors, lawyers, and people with central roles in relevant agencies that our results were consistent with the real employment situation and poverty of patients with HBV. The graphical explanation of the KJ method describes the entire grouping processes from the original 20 labels selected by the multistage pickup without omission. In this grouping, we called the perception of unified label groups “nameplates,” and unified group labels “islands.” The symbols assigned to each island were eventually unified into seven “symbol marks.” In the text, the original labels, last nameplates, and symbol marks are expressed as “”,< >, << >>, respectively.

To verify the statistical relationship between daily life suffering and objective factors (present disease, employment, and income status), we performed binomial logistic regression analysis using daily life suffering as the dependent variable. Daily life suffering was determined by answers to the questionnaire item “What do you think about your everyday life situation?” Two of five possible answers (“I am suffering immensely” and “I am suffering to some extent”) were assigned to the “suffering” group and awarded a score of 1 point. All other answers were awarded 0 points.

For the independent variables and questionnaire, the items were mainly subjective, such as subjective health condition or needs. Therefore, we only analyzed responses to objective questionnaire items that covered patient characteristics. These factors were used as independent variables in analyzing the relationship between subjective daily life suffering and the objective situation. The independent variables for current disease status were asymptomatic carrier, chronic hepatitis, cirrhosis, and liver cancer (yes = 1 point; no = 0 points). The independent variables for current employment status were regular employee (e.g., full-time), non-regular employee (e.g., dispatch worker, contract worker, temporary worker) part-time/casual employee, and unemployed (yes = 1 point; no = 0 points). The analysis of income status showed that all participants’ had incomes below 5,372,000 yen, which was the average income for Japanese households in 2012 according to the 2013 Comprehensive Survey of Living Conditions [25]. The independent income variables were 0–1 million yen (≥0×< 1), 1–2 million yen (≥1×< 2), 2–3 million yen (≥2×< 3), 3–4 million yen (≥3×< 4), 4–5 million yen (≥4×< 5), and 5–6 million yen (≥5×< 6) (yes = 1 point; no = 0 points).

To determine the relationships between the independent variables and the dependent variable (daily life suffering), it was necessary to control for any subjective factors that might influence the dependent variable. Therefore, we incorporated two control independent variables: work-related problems (yes = 1 point; no = 0 points) and debt-related problems (yes = 1 point; no = 0 points). Similarly, we controlled for acceptance of a settlement offer (as this would impact the patient’s financial situation), using the control variable settlement acceptance/non-acceptance (accepted = 1 point; not accepted = 0 points).

In the absence of a previous study showing the above statistical relationships, we used the forced input method to analyze relationships between variables. To analyze the true effects of the variables, we first input the independent variables for current disease status (Model 1), and then added current employment status (Model 2), and finally income status (Model 3).

Furthermore, to prevent multicollinearity issues, we prepared a correlation matrix and checked for correlation coefficients of ±0.9 or more; no such coefficients were observed. All analyses were performed using IBM SPSS Statistics 22.

## Results

### Interview results

**Fig. 1:**
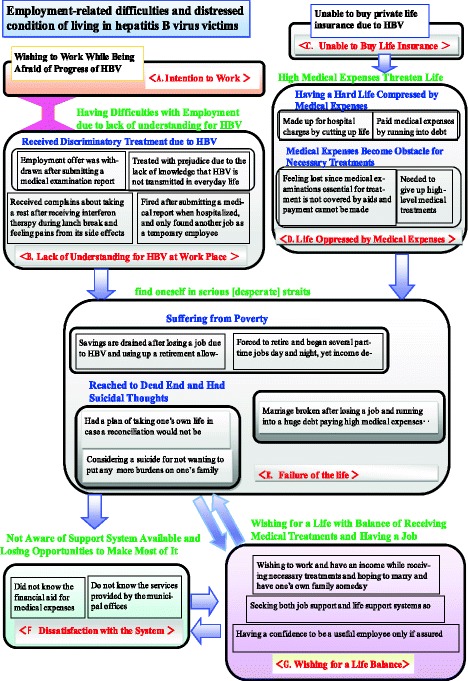
KJ Method Diagram “Employment-related difficulties and ‘distressed condition of living’ in hepatitis B virus victims

After completing two label group formation stages, data were integrated into seven islands. The catchphrases for each island were: intention to work, lack of understanding of HBV in the workplace, inability to buy life insurance, burden due to medical expenses, life failure, dissatisfaction with the system, and wishing for life balance. The completed KJ diagram (“Employment-related difficulties and distressed conditions of living in hepatitis B virus victims”) is presented in Fig. 1. An explanation of each of the seven islands is provided below.

A. **Intention to work**

The interviewees spoke of their desire to work, using language such as **<<**I’m concerned that the I wish to work**>>**. Work was not only perceived as a means of acquiring income, but also referred to as something the interviewee’s liked doing or were motivated to do, conveying their strong determination to work.

B. **Lack of understanding of HBV in the workplace**

Despite their willingness to work, interviewees experienced discriminatory treatment because of HBV. For example, interview participants reported an “employment offer was withdrawn after submitting a medical examination report” and were “treated with prejudice because of a lack of knowledge that HBV cannot be transmitted via daily activities.” They also mentioned that they had “received complaints about taking a rest after receiving interferon therapy during their lunch break and experiencing pain from its side effects.” Furthermore, some were “fired after submitting a medical report when hospitalized, and only found another job as a temporary employee.” These statements suggested that participants **<<**experienced difficulties with employment because of a lack of understanding about HBV**>>** .

C. **Inability to buy life insurance**

The interviewees reported they experienced a disadvantaged financial position if, for example, they were **<<**unable to buy private life insurance because of their HBV diagnosis**> >** .

D. **Burden due to medical expenses**

The interviewees mentioned that they <had a hard life overwhelmed by medical expenses>. For example, they “paid for hospital expenses by making cutbacks in other areas” and “paid medical expenses by going into debt.” The interviewees also noted that <medical expenses prevented necessary treatments>. For example, they reported “feeling hopeless because essential medical examinations are not covered by aid and they could not make payments,” and “needed to give up high-level medical treatment.” These statements show how the interview participants struggled to live a normal life because their **<<**high medical expenses impacted on their lifestyle**>>** .

E. **Life failure**

Because of their disease, interviewees reported they were financially crippled or <<found themselves in serious, desperate straits>>. For example, “savings were drained after losing their jobs because of HBV, and they were spending retirement allowances to pay for medical expenses,” and they were “forced to retire and begin several part-time day and night jobs; yet, their income had declined by one-thirds.”

In such circumstances, some interviewees faced the problem of “marriages ending after losing jobs and incurring significant debt to pay high medical expenses.” This indicates that some interviewees were financially ruined by high medical expenses, as their income was reduced after losing their job because of their disease. In turn, this led to relationship breakups with significant others.

The interviews also highlighted participants’ desperation. For example, many “had a suicide plan in case a government settlement would not be reached in court” or were “considering suicide so as not to place further burdens on family.”

F. **Dissatisfaction with the system**

One interviewee was <<not aware of available support systems and was losing opportunities to make the most of them>>. Furthermore, other interviewees “did not know about financial aid for medical expenses” and “did not know about the services provided by municipal offices.”

G. **Wishing for life balance**

Interview participants reported that they **<<**wished for life balance regarding medical treatments and work**>>**. For example, they “wished to work and have an income while receiving necessary treatments, and hoped to marry and have their own families someday,” “wanted to be a useful employee, but only if they could also receive medical treatment and keep working,” and “sought both job support and life support systems to achieve an independent and decent life.”

### Binomial logistic regression analysis

The binomial logistic regression analysis with daily life suffering as the dependent variable showed that for current disease status (Model 1), there were significant positive correlations with cirrhosis (odds ratio [OR] 1.47, 95% confidence interval [CI]: 1.02–2.12, *p* < 0.05) and liver cancer (OR 1.56, 95% CI: 1.09–2.25, *p* < 0.05).

When we added current employment status to the model (Model 2), we observed significant positive correlations with cirrhosis (OR 1.47, 95% CI: 1.01–2.13, *p* < 0.05) and liver cancer (OR 1.44, 95% CI: 1.00–2.08, *p* < 0.05). We also observed a significant negative correlation with being a regular employee (OR 0.53, 95% CI: 0.42–0.66 *p* < 0.01), and significant positive correlations with being a part-time/casual employee (OR 1.52, 95% CI: 1.18–1.95 *p* < 0.01) or unemployed (OR 1.47, 95% CI: 1.19–1.82 *p* < 0.01).

In the model with both current employment status and income status (Model 3), we observed significant positive correlations between daily life suffering and liver cancer (OR 1.47, 95% CI: 1.00–2.17, *p* < 0.05), being a part-time/casual employee (OR 1.46, 95% CI: 1.11–1.92 *p* < 0.01), and all income increments from 0 to 1 million yen to 5–6 million yen (*p* < 0.01). In addition, the OR was inversely proportional to annual income (OR 31.12–5.17; see Table 2).

**Table 2:** Results of the binomial logistic regression analysis on the factors related to suffering among HBV victims

Factors	Responses	Present disease status		Present employment status		Income status	
		OR	95% CI	OR	95% CI	OR	95% CI
Age		0.98	0.95–1.01	0.98	0.98–0.99^**^	0.97	0.96–0.98^***^
Sex	Male	0.98	0.84–1.15	1.13	0.95–1.33	1.16	0.97–1.39
	Female	1.00		1.00		1.00	
Settlement acceptance	Accepted	0.71	0.61–0.82^***^	0.70	0.60–0.81^***^	0.69	0.59–0.81^***^
	Not accepted	1.00		1.00		1.00	
Work-related problems	Yes	0.94	0.80–1.10	1.11	0.93–1.31	1.05	0.88–1.26
	No	1.00		1.00		1.00	
Debt-related problems	Yes	6.76	5.77–7.91^***^	6.39	5.44–7.49^***^	5.78	4.88–6.85^***^
	No	1.00		1.00		1.00	
Asymptomatic carrier	Yes	1.35	0.91–2.00	1.33	0.89–1.99	1.45	0.95–2.22
	No	1.00		1.00			
Chronic hepatitis	Yes	1.42	0.98–2.07	1.43	0.98–2.09	1.47	0.99–2.20
	No	1.00		1.00			
Cirrhosis	Yes	1.47	1.02–2.12^*^	1.47	1.01–2.13*	1.47	0.99–2.17
	No	1.00		1.00			
Liver cancer	Yes	1.56	1.09–2.25^*^	1.44	1.00–2.08*	1.47	1.00–2.17*
	No	1.00		1.00			
Regular employee (full-time, etc.)	Yes			0.53	0.42–0.66^***^	0.90	0.71–1.15
	No			1.00			
Non-regular employee (dispatch, contract, temporary worker, etc.)	Yes			1.01	0.75–1.35	0.96	0.70–1.32
	No			1.00			
Part-time/casual employee	Yes			1.52	1.18–1.95^***^	1.46**	1.11–1.92
	No			1.00			
Unemployed	Yes			1.47	1.19–1.82^***^	1.03	0.82–1.30
	No			1.00			
0–1 million yen	Yes					31.12	19.2–50.2^***^
	No					1.00	
1–2 million yen	Yes					29.83	19.8–44.9^***^
	No					1.00	
2–3 million yen	Yes					16.76	11.4–24.6^***^
	No					1.00	
3–4 million yen	Yes					7.77	5.32–11.3***
	No					1.00	
4–5 million yen	Yes					5.45	3.68–8.08^***^
	No					1.00	
5–6 million yen	Yes					5.17	3.46–7.74^***^
	No					1.00	
Model *Χ* ^2^ (*df*)		722.2 (9)^***^		812.8 (13)***		1280.6 (19)^***^	

*OR* odds ration, *CI* confidence interval; ^***^ *p* < .001; ^**^ *p* < .01; ^*^ *p* < .05.

## Discussion

### Effect of current disease status on daily life suffering: Medical expenses

In 2004, at least nine international studies reported on the costs associated with treating HBV [26], and showed that medical costs are a significant burden for patients. Our qualitative study confirmed that patients with HBV face a worsening disease state, declining income resulting from employment-related difficulties, and increasing medical expenses. As there is presently no cure for HBV, patients face a long-term struggle with the disease. Consequently, many patients bear significant medical costs, as highlighted in the label “my medical costs were around 20 million yen.” Such costs include frequent hospital visits, living-donor liver transplants not covered by insurance, and the unavoidable use of private cubicles (before the introduction of Japan’s medical expenses subsidy system). Other medical costs besides consultation and medicine include examination, travel, and hospitalization expenses (e.g., food and pajamas). Normally, private insurance provides support in such emergencies, but in many cases, patients with HBV were unable to take out or renew policies because “hepatitis is not covered.” Consequently, medical expenses had a major impact on patients’ lives, as highlighted by the label “paid for hospital expenses by making cutbacks in other areas.” Increased medical costs due to deterioration of disease state and decreased income due to employment difficulties occurred simultaneously. The long-term struggle of many patients resulted in “danger to life and existence.”

Our qualitative study showed an association between the severity of cirrhosis or liver cancer and the probability of experiencing suffering in daily life. The increased probability of experiencing suffering in daily life after symptoms of HBV intensify may be natural. However, these severe symptoms may confer physical and financial burdens and increase daily life suffering. Although hepatitis, including chronic hepatitis, qualifies patients for medical subsidies, circumstances change when the disease progresses to cirrhosis or liver cancer; therefore, patients “feel hopeless because essential medical examinations are not covered by aid and the inability to pay.” Some patients “paid for medical expenses by going into debt,” sold houses or farmland to pay expenses, or decided to give up treatment (“needed to give up high-level medical treatments”).

The effect of income was no longer shown in Model 3, with the significant positive correlation with HBV seen in Model 1 not found. Further, the OR for liver cancer was reduced. However, these statistical relationships indicate correlations, rather than direct causal relationships. Cases that indicated direct causal relationships in the interview data may be used to explain these results from a medical expense perspective. Cirrhosis and liver cancer increased the requirements for long-term hospitalization and advanced anticancer therapies not covered by insurance. In these cases, disease status might have increased the likelihood of daily life suffering by increasing the financial burden. Therefore, our quantitative results are consistent with those of our qualitative study, and confirm their validity.

### Influence of current employment status on daily life suffering

Our results showed that patients with HBV were demoted or forced to quit their jobs because of: 1) deterioration of physical condition due to disease progression, 2) decrease in working hours due to outpatient or inpatient treatment, or 3) discriminatory treatment based on prejudice or misunderstanding. When regularly employed people become irregularly employed, their income significantly decreases; if they eventually become unemployed, it leads to further deterioration.

The interviews revealed that some patients with HBV wished to work but faced barriers to securing employment, including: 1) deteriorating physical condition concomitant to disease progression; 2) reduction of working hours because of hospital commutes and time spent in the hospital; and 3) discriminatory treatment because of prejudice and misconceptions. Therefore, they “made an effort to continue work while receiving treatment, but failed to foster understanding in their workplaces,” and consequently, felt pressured to accept demotion or dismissal. This represents disease-attributed downscaling of employment status (e.g., “fired after submitting a medical report when hospitalized, and only finding another job as a temporary employee”). Patients then faced a dramatic decline in income, for example, being “forced to retire and begin several part-time day and night jobs; yet, my income has declined by two-thirds” or “receiving 100,000 yen in pay from a part-time job, which comes to 40,000 after subtracting national insurance, pension contributions, and medical expenses; this is not enough to live on.” Eventually, as their disease progressed, some left work altogether, resulting in crisis (e.g., “at wits end; nothing else to do but to receive advance pension payments and accept a benefit level at 70% of the normal amount”).

Our quantitative study indicated that the likelihood of daily life suffering increased as employment status moved from regular to less secure (→ “part-time/casual employee” → “unemployed”); this finding is consistent with the situation described in the interviews. This forced acceptance of changes in employment status and consequent crises was observed in the entire study sample.

### Influence of income status on daily life suffering

The analysis with patients whose incomes were lower than the national per capita income indicated that lower annual income was associated with an elevated chance of experiencing daily life suffering. This group was forced into less stable jobs (reflected in their reduced income), while their medical expenses were higher than those of healthy individuals. Therefore, this association between income status and daily life suffering difficulties is characteristic of patients with HBV.

Our qualitative study indicated that the specific causal factor of daily life suffering represented by “current disease status” was the increase in medical costs owing to disease progression, and reflects household budget expenditure. The specific causal factor of daily life suffering represented by “current employment status” was the wage reduction attributable to employment insecurity because of HBV. Our quantitative results for participants with incomes below the 2012 average per capita income (Comprehensive Survey of Living Conditions) indicated that the likelihood of daily life suffering increased as annual income decreased. Among patients with HBV, this relationship should be discussed with reference to increased medical expenditure and decreased wages following the shift to less secure employment because of their HBV diagnosis.

This relationship between income status and daily life suffering does not mean that daily life suffering occurs simply because of earning a low income. Any interpretation of this relationship should be based on the understanding that the income status of patients with HBV is related to their increasingly insecure employment status, and medical costs account for a greater ratio of their household expenditure compared with those of healthy individuals. Therefore, the relationship between income status and daily life suffering, as described in this study, is not simply a general trend but one particular to patients with HBV.

### Prejudice and discrimination related to HBV and treatment

As highlighted, discrimination and discriminatory treatment stemming from misunderstanding and prejudice (also referred to as stigma) is problematic in an employment context. When HIV was first reported in the Japanese media, people living with HIV suffered from open stigmatization. Although there have been reports in the media about HCV, there has been relatively little coverage of HBV; however, we found reports of discrimination from people with HBV, especially regarding employment.

Ellard and Wallace [27] from Australia’s La Trobe University published a literature review of stigma, discrimination, and HBV based on 39 studies. They observed that “Evidence suggests that when stigma and discrimination are associated with people with chronic hepatitis B it is primarily related to a poor understanding of prevention and transmission, and not linked to concepts of moral deficit that characterize HIV- and hepatitis C-related stigma and discrimination” (p. 2). To reduce HBV-related stigma and discrimination, they recommend “developing an understanding of any relationship between levels and quality of knowledge about hepatitis B, and stigma and discrimination” in future research (p. 4). As those authors suggested, we need to clarify the understanding of those who are prejudiced and discriminate about HBV and the related stigma.

A countermeasure identified in the 2009 Basic Act on Hepatitis Measures and established by the Japanese Ministry of Health, Labour and Welfare was a research group. The group was formed to better understand the prejudice and discrimination directed toward patients with hepatitis and to create guidelines for harm prevention. The group conducted research from 2011 to 2013, and published their results annually. Tatsuoka [28] summarized these results in a 15-page editorial. This proposed “measures to prevent prejudice and discrimination against hepatitis patients” including: 1) viral hepatitis medication and treatment development; 2) public relations activities and education to raise awareness and spread accurate knowledge about viral hepatitis transmission and treatment; 3) promotion of general education regarding prejudice and discrimination as a complement to these core activities; and 4) defining direct and indirect systemic roles.

Such measures should be based on an understanding of current needs and context, including a system to receive complaints and requests for advice about prejudice and discrimination, and systems for the prevention of, recovery from, and sanctions against victimization. For national level anti-discrimination systems, the report notes that each country’s need for systems of some kind stems from its particular circumstances and historical background. For example, the US has a Federal Americans with Disabilities Act, the UK, Germany, and other countries have anti-discrimination laws, South Korea has a National Human Rights Commission, and Sweden has an Equality Ombudsman.

### Distressed living conditions

Patients with HBV in our study suffered from employment difficulties due to deterioration of disease condition and extreme poverty induced by medical costs, as well as: 1) severing of ties (disruption of family economic situation due to factors such as unemployment and medical costs); 2) life-threatening crises (severity of condition as a result of giving up treatments because of financial difficulties); and, 3) loss of the will to live (considering suicide because of poverty and deterioration of family relationships). These types of severe damage, along with economic hardships, may negatively transform people’s existence and ruin their lives, and are serious issues.

A study on individuals with subacute myelo-optic neuropathy (an iatrogenic disease caused by the drug clioquinol) clarified the types of medicine-attributed damage they suffered; namely physical, psychological, financial, and social damage [29]. A fact-finding study of victims of iatrogenic HCV transmission raised similar concerns [23]. In addition, along with the reconciliation of the lawsuit in June 2011, won by efforts from the plaintiff group and its counsel, victims affected by HBV due to group vaccination are now able to receive some compensation, depending on the conditions after official recognition as a victim. Therefore, these victims should experience a less burdened life after receiving compensation in terms of “poverty in living”.However, were only able to include control variables for “whether reconciliation was made,” and it was not possible to analyze the gap in living situations before/after compensation was paid in those who answered “yes” to the question “whether payment for reconciliation was made.” We would like to examine this point in a future study.

### Urgent need to establish and implement measures to assist patients with HBV

In March 2015, the WHO issued “Guidelines for the prevention, care, and treatment of persons with chronic hepatitis B” [30], as an estimated 240 million patients had chronic HBV worldwide. Although the WHO’s direct approach to addressing the problem can be appreciated, those guidelines focus on treatment with antiviral medications and the prevention of further infection (preventing transmission). The guidelines do not address the economic and social issues discussed in our study (i.e., living and treatment expenses and employment-related prejudice and discrimination).

In 2008, the WHO Commission on Social Determinants of Health issued its final report, “Closing the gap in a generation: health equity through action on the social determinants of health” [30]. This report takes a holistic view of the social determinants of health. Based on that evidence, M. Marmot, the chairman of that committee, described the “proportionate universalism,” [31] which represents making social welfare systems proportionately more robust for people in the level of disadvantage (e.g., a lower annual income or weaker employment situation) while preserving the universal natuer of social intervations. This idea is consistent with the findings of the present study that daily life suffering experienced by patients with HBV is highly prevalent and associated with lower wages and increased expenses.

Problems concerning medical treatment, the social and economic problems of employment assistance, and the elimination of prejudice and discrimination are all pressing issues. Starting with Japan’s Basic Act on Hepatitis Measures, national governments and international institutions (e.g., the WHO) should address the issues surrounding HBV infection through this kind of awareness.

### Contribution and limitations

For our quantitative study, we selected 11,046 participants who had been infected with HBV through group vaccinations and had jointly filed lawsuits in District Courts before 2014. We obtained 6525 responses (response rate: 60.1%). Using both qualitative and quantitative analyses, we clarified the employment-related hardships experienced by patients with HBV, and determined the relationship between their daily life suffering and their subsequent descent into poverty. A 2015 study with 1311 patients with HBV in Japan conducted by the Ministry of Health, Labour and Welfare did not evaluate this relationship. As it is estimated that more than 400,000 people have been infected with HBV by various means, including group vaccinations, we were unable to interview all infected persons.

Our study used self-report data, and it is likely that those with greater stigmatization/financial burden experienced a greater degree of suffering and might have been more likely to respond (i.e., self-report bias). Another limitation of our study is the binary nature of the quantitative variables and the subjective nature of their formation, which might have resulted in measurement error and information loss.

## Conclusions

We demonstrated that people with HBV experience daily life suffering associated with employment-related hardships. Their likelihood of experiencing distress in everyday life increased in proportion to: 1) increasing medical expenses accompanying deterioration of their condition; 2) increasingly insecure employment status attributable to HBV; and 3) the resulting decrease in income. In addition, prejudice and discrimination had an impact on these relationships.

Considering the results of the present study and previous studies, the daily life suffering experienced by patients with HBV is highly prevalent and associated with employment-related hardships (e.g., lower wages) and increases in expenses, and is characterized by distressed living conditions. Patients with HBV need social support to help eliminate prejudice and discrimination, and consideration of their social vulnerability based on the idea of “proportional universalism.”

## Abbreviations

HBV: Hepatitis B virus
HCV: Hepatitis C virus
HIV: Human immunodeficiency virus
KJ Method: Kawakita Jiro Method (developed in 1986)
WHO: World Health Organization
